# Methyl (2*Z*)-3-[(4-nitro­phen­yl)carbamo­yl]prop-2-enoate

**DOI:** 10.1107/S1600536810050956

**Published:** 2010-12-11

**Authors:** Khurram Shahzad Munawar, Saqib Ali, M. Nawaz Tahir

**Affiliations:** a Department of Chemistry, Quaid-e-Azam University, Islamabad, Pakistan; b Department of Physics, University of Sargodha, Sargodha, Pakistan

## Abstract

In the title compound, C_11_H_10_N_2_O_5_, the amide group is nearly coplanar and the ester group approximately perpendicular to the vinyl C—HC=CH—C group [dihedral angles of 5.0 (2) and 88.89 (5)°, respectively]. This results in a short intra­molecular O =C⋯O=C contact of 2.7201 (17) Å between the amide O atom and the ester carbonyl C atom. The prop-2-enamide fragment and the nitro group make dihedral angles of 20.42 (6) and 13.54 (17)°, respectively, with the benzene ring. An intra­molecular C—H⋯O inter­action between the benzene ring and the amide group generates an *S*(6) ring motif. Inter­molecular C—H⋯O and N—H⋯O hydrogen bonds complete *R*_2_^2^(11) ring motifs and join mol­ecules into [100] chains.

## Related literature

For crystal structures of *N*-substituted maleamic acids, see: Lo & Ng (2009 ▶); Wardell *et al.* (2005 ▶). For the synthesis of (4-[(4-nitro­phen­yl)amino]-4-oxobut-2-enoic acid, see: Shahid *et al.* (2006 ▶). For graph-set notation, see: Bernstein *et al.* (1995 ▶).
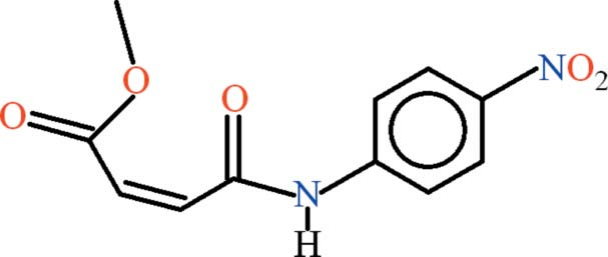


## Experimental

### 

#### Crystal data

C_11_H_10_N_2_O_5_
*M* *_r_* = 250.21
Triclinic, 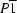

*a* = 6.8382 (2) Å
*b* = 7.7497 (2) Å
*c* = 11.8277 (5) Å
α = 97.805 (2)°
β = 92.119 (2)°
γ = 114.425 (1)°
*V* = 562.39 (3) Å^3^
*Z* = 2
Mo *K*α radiation
μ = 0.12 mm^−1^
*T* = 296 K
0.35 × 0.26 × 0.24 mm

#### Data collection

Bruker Kappa APEXII CCD diffractometer
Absorption correction: multi-scan (*SADABS*; Bruker, 2005 ▶) *T*_min_ = 0.897, *T*_max_ = 0.922
8150 measured reflections
2021 independent reflections
1754 reflections with *I* > 2σ(*I*)
*R*_int_ = 0.020

#### Refinement

*R*[*F* ^2^ > 2σ(*F* ^2^)] = 0.035
*wR*(*F* ^2^) = 0.097
*S* = 1.08
2021 reflections
164 parameters
H-atom parameters constrained
Δρ_max_ = 0.14 e Å^−3^
Δρ_min_ = −0.15 e Å^−3^

### 

Data collection: *APEX2* (Bruker, 2009 ▶); cell refinement: *SAINT* (Bruker, 2009 ▶); data reduction: *SAINT*; program(s) used to solve structure: *SHELXS97* (Sheldrick, 2008 ▶); program(s) used to refine structure: *SHELXL97* (Sheldrick, 2008 ▶); molecular graphics: *ORTEP-3 for Windows* (Farrugia, 1997 ▶) and *PLATON* (Spek, 2009 ▶); software used to prepare material for publication: *WinGX* (Farrugia, 1999 ▶) and *PLATON*.

## Supplementary Material

Crystal structure: contains datablocks global, I. DOI: 10.1107/S1600536810050956/gk2328sup1.cif

Structure factors: contains datablocks I. DOI: 10.1107/S1600536810050956/gk2328Isup2.hkl

Additional supplementary materials:  crystallographic information; 3D view; checkCIF report

## Figures and Tables

**Table 1:** Hydrogen-bond geometry (Å, °)

*D*—H⋯*A*	*D*—H	H⋯*A*	*D*⋯*A*	*D*—H⋯*A*
N1—H1⋯O2^i^	0.86	2.11	2.9467 (17)	164
C7—H7⋯O3	0.93	2.33	2.8983 (17)	119
C11—H12⋯O3^i^	0.93	2.45	3.3020 (19)	152

Symmetry code: (i) 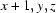.

**Fig. 1.**
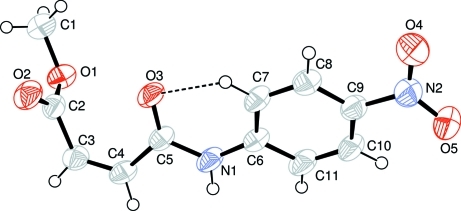
View of the title compound with the atom numbering scheme. The displacement ellipsoids are drawn at the 50% probability level. H atoms are shown by small circles of arbitrary radii. The dotted line shows intramolecular hydrogen bond.

**Fig. 2.**
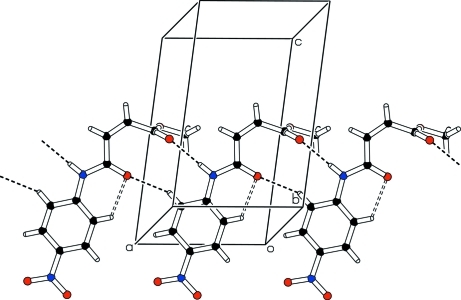
The partial packing (PLATON; Spek, 2009) which shows one dimensional polymeric chain of hydrogen-bonded molecules, extending along the a-axis.

## supplementary crystallographic information

### Comment

The title compound (I, Fig. 1) has been crystallized in an attempt to synthesize the vanadium complex of 3-(4-nitrophenylaminocarbonyl)prop-2-enoic acid.

The crystal structure of *N*-phenylmaleamic acid (II) (Lo & Ng, 2009) and (*E*)-3-(4-nitrophenylaminocarbonyl)prop-2-enoic acid (III) (Wardell *et al.*, 2005) have been published which are related to (I).

In (I), the methyl formate and prop-2-enamide moieties A (C1/O1/C2/O2) and B (C3/C4/C5/N1/O3) are planar with r. m. s. deviations of 0.012 and 0.019 Å, respectively. The benzene ring C (C6—C11) is planar with r. m. s. deviation of 0.008 Å. The nitro group D (N2/O4/O5) is of course planar. The dihedral angle between A/B, A/C, A/D, B/C, B/D and C/D is 88.78 (4), 86.03 (5), 80.82 (14), 20.42 (6), 12.62 (20) and 13.54 (17)°, respectively. In (I) the value of C═C is 1.318 (2) Å. There exists an intramolecular hydrogen bonding of C—H···O type (Table 1, Fig. 1) completing an S(6) ring motif (Bernstein *et al.*, 1995). There exist intermolecular hydrogen bondings of C—H···O and N—H···O types (Table 1, Fig. 2). Due to these H-bondings *R*_2_^2^(11) ring motifs are formed and the molecules are finally stabilized in the form of one dimensional polymeric chains extending along the crystallographic *a* axis (Fig. 2).

### Experimental

3-(4-Nitrophenylaminocarbonyl)prop-2-enoic acid was prepared according to the procedure reported by Shahid *et al.* (2006). 3-(4-Nitrophenylaminocarbonyl)prop-2-enoic acid (3 mmol) and VCl_3_ (1 mmol) were refluxed in methanol for 4 h resulting in greenish solution. Light green prisms of the title compound were formed after two days.

### Refinement

The H-atoms were positioned geometrically (N—H = 0.86, C–H = 0.93–0.96 Å) and treated as riding with *U*_iso_(H) = *xU*_eq_(C, N), where *x* = 1.5 for methyl and *x* = 1.2 for all other H-atoms.

### Crystal data

**Table:**

C_11_H_10_N_2_O_5_	*Z* = 2
*M**_r_* = 250.21	*F*(000) = 260
Triclinic, *P*1	*D*_x_ = 1.478 Mg m^−^^3^
Hall symbol: -P 1	Mo *K*α radiation, λ = 0.71073 Å
*a* = 6.8382 (2) Å	Cell parameters from 1754 reflections
*b* = 7.7497 (2) Å	θ = 3.2–25.3°
*c* = 11.8277 (5) Å	µ = 0.12 mm^−^^1^
α = 97.805 (2)°	*T* = 296 K
β = 92.119 (2)°	Prism, light green
γ = 114.425 (1)°	0.35 × 0.26 × 0.24 mm
*V* = 562.39 (3) Å^3^	

### Data collection

**Table:**

Bruker Kappa APEXII CCD diffractometer	2021 independent reflections
Radiation source: fine-focus sealed tube	1754 reflections with *I* > 2σ(*I*)
graphite	*R*_int_ = 0.020
Detector resolution: 8.10 pixels mm^-1^	θ_max_ = 25.3°, θ_min_ = 3.2°
ω scans	*h* = −8→8
Absorption correction: multi-scan (*SADABS*; Bruker, 2005)	*k* = −9→9
*T*_min_ = 0.897, *T*_max_ = 0.922	*l* = −14→14
8150 measured reflections	

### Refinement

**Table:**

Refinement on *F*^2^	Primary atom site location: structure-invariant direct methods
Least-squares matrix: full	Secondary atom site location: difference Fourier map
*R*[*F*^2^ > 2σ(*F*^2^)] = 0.035	Hydrogen site location: inferred from neighbouring sites
*wR*(*F*^2^) = 0.097	H-atom parameters constrained
*S* = 1.08	*w* = 1/[σ^2^(*F*_o_^2^) + (0.0475*P*)^2^ + 0.101*P*] where *P* = (*F*_o_^2^ + 2*F*_c_^2^)/3
2021 reflections	(Δ/σ)_max_ < 0.001
164 parameters	Δρ_max_ = 0.14 e Å^−^^3^
0 restraints	Δρ_min_ = −0.15 e Å^−^^3^

### Special details

**Table:**

Geometry. Bond distances, angles *etc*. have been calculated using the rounded fractional coordinates. All su's are estimated from the variances of the (full) variance-covariance matrix. The cell e.s.d.'s are taken into account in the estimation of distances, angles and torsion angles
Refinement. Refinement of *F*^2^ against ALL reflections. The weighted *R*-factor *wR* and goodness of fit *S* are based on *F*^2^, conventional *R*-factors *R* are based on *F*, with *F* set to zero for negative *F*^2^. The threshold expression of *F*^2^ > σ(*F*^2^) is used only for calculating *R*-factors(gt) *etc*. and is not relevant to the choice of reflections for refinement. *R*-factors based on *F*^2^ are statistically about twice as large as those based on *F*, and *R*- factors based on ALL data will be even larger.

### Fractional atomic coordinates and isotropic or equivalent isotropic displacement parameters (Å^2^)

**Table:**

	*x*	*y*	*z*	*U*_iso_*/*U*_eq_	
O1	0.14198 (15)	0.84577 (15)	0.41306 (9)	0.0545 (3)	
O2	0.10967 (16)	1.06232 (16)	0.31587 (9)	0.0568 (4)	
O3	0.34399 (14)	0.84813 (15)	0.17681 (8)	0.0518 (3)	
O4	0.5882 (2)	0.46462 (19)	−0.33243 (11)	0.0766 (5)	
O5	0.92706 (19)	0.55754 (18)	−0.28426 (12)	0.0743 (5)	
N1	0.70053 (17)	0.91905 (18)	0.16737 (10)	0.0484 (4)	
N2	0.7481 (2)	0.54311 (18)	−0.26298 (12)	0.0567 (5)	
C1	−0.0900 (3)	0.7351 (3)	0.39699 (17)	0.0670 (6)	
C2	0.2191 (2)	1.0025 (2)	0.36544 (11)	0.0433 (4)	
C3	0.4580 (2)	1.1052 (2)	0.39010 (12)	0.0467 (4)	
C4	0.5981 (2)	1.0757 (2)	0.32569 (12)	0.0467 (4)	
C5	0.5316 (2)	0.9364 (2)	0.21728 (12)	0.0422 (4)	
C6	0.6983 (2)	0.8127 (2)	0.06154 (12)	0.0429 (4)	
C7	0.5276 (2)	0.7463 (2)	−0.02453 (12)	0.0461 (5)	
C8	0.5423 (2)	0.6543 (2)	−0.12949 (12)	0.0476 (4)	
C9	0.7270 (2)	0.6289 (2)	−0.14903 (13)	0.0466 (4)	
C10	0.8956 (2)	0.6898 (2)	−0.06399 (14)	0.0542 (5)	
C11	0.8810 (2)	0.7806 (2)	0.04099 (14)	0.0537 (5)	
H1	0.82501	0.98188	0.20614	0.0581*	
H1A	−0.15939	0.81926	0.41485	0.1005*	
H1B	−0.13094	0.64057	0.44679	0.1005*	
H1C	−0.13336	0.67204	0.31868	0.1005*	
H3	0.51277	1.19737	0.45606	0.0560*	
H4	0.74467	1.14518	0.34934	0.0560*	
H7	0.40353	0.76407	−0.01099	0.0553*	
H8	0.42826	0.60944	−0.18708	0.0571*	
H11	1.01806	0.66940	−0.07773	0.0651*	
H12	0.99377	0.82118	0.09894	0.0644*	

### Atomic displacement parameters (Å^2^)

**Table:**

	*U*^11^	*U*^22^	*U*^33^	*U*^12^	*U*^13^	*U*^23^
O1	0.0396 (5)	0.0613 (7)	0.0579 (6)	0.0170 (5)	0.0001 (4)	0.0103 (5)
O2	0.0425 (6)	0.0708 (7)	0.0618 (7)	0.0301 (5)	−0.0034 (5)	0.0087 (5)
O3	0.0288 (5)	0.0674 (7)	0.0542 (6)	0.0187 (5)	−0.0003 (4)	0.0006 (5)
O4	0.0659 (8)	0.0831 (9)	0.0654 (8)	0.0238 (7)	0.0026 (6)	−0.0111 (7)
O5	0.0610 (7)	0.0715 (8)	0.0904 (9)	0.0299 (6)	0.0290 (6)	0.0013 (7)
N1	0.0273 (5)	0.0678 (8)	0.0473 (7)	0.0184 (5)	−0.0002 (4)	0.0069 (6)
N2	0.0524 (8)	0.0481 (7)	0.0670 (9)	0.0186 (6)	0.0162 (7)	0.0070 (6)
C1	0.0427 (9)	0.0682 (11)	0.0792 (12)	0.0157 (8)	0.0089 (8)	0.0018 (9)
C2	0.0382 (7)	0.0559 (8)	0.0360 (7)	0.0230 (6)	0.0007 (5)	−0.0013 (6)
C3	0.0389 (7)	0.0549 (8)	0.0425 (7)	0.0184 (6)	−0.0043 (5)	0.0032 (6)
C4	0.0312 (7)	0.0570 (8)	0.0477 (8)	0.0145 (6)	−0.0022 (5)	0.0103 (6)
C5	0.0318 (7)	0.0542 (8)	0.0432 (7)	0.0194 (6)	0.0025 (5)	0.0134 (6)
C6	0.0301 (6)	0.0509 (8)	0.0476 (8)	0.0154 (6)	0.0051 (5)	0.0133 (6)
C7	0.0318 (7)	0.0601 (9)	0.0507 (8)	0.0225 (6)	0.0045 (5)	0.0130 (7)
C8	0.0369 (7)	0.0543 (8)	0.0500 (8)	0.0175 (6)	0.0005 (6)	0.0100 (7)
C9	0.0399 (7)	0.0435 (8)	0.0553 (8)	0.0151 (6)	0.0115 (6)	0.0110 (6)
C10	0.0334 (7)	0.0630 (9)	0.0693 (10)	0.0233 (7)	0.0100 (7)	0.0101 (8)
C11	0.0306 (7)	0.0705 (10)	0.0599 (9)	0.0217 (7)	0.0005 (6)	0.0103 (7)

### Geometric parameters (Å, °)

**Table:**

O1—C1	1.448 (2)	C6—C7	1.392 (2)
O1—C2	1.3224 (18)	C7—C8	1.374 (2)
O2—C2	1.2029 (19)	C8—C9	1.379 (2)
O3—C5	1.2177 (18)	C9—C10	1.378 (2)
O4—N2	1.220 (2)	C10—C11	1.370 (2)
O5—N2	1.221 (2)	C1—H1A	0.9600
N1—C5	1.363 (2)	C1—H1B	0.9600
N1—C6	1.3980 (18)	C1—H1C	0.9600
N2—C9	1.459 (2)	C3—H3	0.9300
N1—H1	0.8600	C4—H4	0.9300
C2—C3	1.488 (2)	C7—H7	0.9300
C3—C4	1.318 (2)	C8—H8	0.9300
C4—C5	1.480 (2)	C10—H11	0.9300
C6—C11	1.395 (2)	C11—H12	0.9300
			
O1···O3	3.1615 (14)	C1···C2^ix^	3.595 (2)
O1···O5^i^	3.1218 (17)	C2···O3	2.7201 (17)
O1···C3^ii^	3.3877 (18)	C2···C1^ix^	3.595 (2)
O1···C4^ii^	3.3565 (18)	C3···O1^ii^	3.3877 (18)
O2···C9^iii^	3.1785 (18)	C3···C3^ii^	3.400 (2)
O2···O3	3.1114 (16)	C4···O1^ii^	3.3565 (18)
O2···N1^iv^	2.9467 (17)	C5···O4^i^	3.363 (2)
O2···N2^iii^	2.9652 (17)	C6···C8^i^	3.523 (2)
O2···O5^iii^	3.1282 (18)	C7···O3	2.8983 (17)
O3···O1	3.1615 (14)	C8···C6^i^	3.523 (2)
O3···C11^iv^	3.3020 (19)	C9···O2^iii^	3.1785 (18)
O3···C7	2.8983 (17)	C11···C11^vii^	3.404 (2)
O3···O2	3.1114 (16)	C11···O3^viii^	3.3020 (19)
O3···C2	2.7201 (17)	C2···H1A^ix^	2.9000
O3···N2^i^	3.1522 (17)	C5···H7	2.7800
O4···C5^i^	3.363 (2)	H1···O2^viii^	2.1100
O4···C1^v^	3.116 (3)	H1···H4	2.2000
O5···O1^i^	3.1218 (17)	H1···H12	2.3100
O5···O2^iii^	3.1282 (18)	H1A···O2	2.4900
O5···C1^i^	3.089 (3)	H1A···O4^v^	2.8700
O1···H4^ii^	2.8700	H1A···C2^ix^	2.9000
O2···H1C	2.7900	H1C···O2	2.7900
O2···H1A	2.4900	H1C···O4^v^	2.8600
O2···H12^iv^	2.8300	H1C···O5^i^	2.6900
O2···H1^iv^	2.1100	H1C···H8^v^	2.5400
O2···H4^iv^	2.8500	H3···O4^x^	2.9000
O3···H12^iv^	2.4500	H4···O2^viii^	2.8500
O3···H7	2.3300	H4···H1	2.2000
O4···H1A^v^	2.8700	H4···O1^ii^	2.8700
O4···H1C^v^	2.8600	H4···O5^vii^	2.7000
O4···H3^vi^	2.9000	H7···O3	2.3300
O4···H8	2.4600	H7···C5	2.7800
O5···H11	2.4400	H7···H11^iv^	2.4900
O5···H1C^i^	2.6900	H8···O4	2.4600
O5···H4^vii^	2.7000	H8···H1C^v^	2.5400
N1···O2^viii^	2.9467 (17)	H11···O5	2.4400
N2···O2^iii^	2.9652 (17)	H11···H7^viii^	2.4900
N2···O3^i^	3.1522 (17)	H12···O2^viii^	2.8300
C1···O4^v^	3.116 (3)	H12···O3^viii^	2.4500
C1···O5^i^	3.089 (3)	H12···H1	2.3100
			
C1—O1—C2	116.39 (13)	C8—C9—C10	121.14 (14)
C5—N1—C6	128.61 (13)	C9—C10—C11	119.34 (14)
O4—N2—O5	123.51 (15)	C6—C11—C10	120.46 (14)
O4—N2—C9	118.66 (14)	O1—C1—H1A	109.00
O5—N2—C9	117.81 (14)	O1—C1—H1B	109.00
C5—N1—H1	116.00	O1—C1—H1C	109.00
C6—N1—H1	116.00	H1A—C1—H1B	109.00
O2—C2—C3	124.08 (13)	H1A—C1—H1C	109.00
O1—C2—O2	124.52 (14)	H1B—C1—H1C	109.00
O1—C2—C3	111.18 (12)	C2—C3—H3	117.00
C2—C3—C4	125.07 (13)	C4—C3—H3	117.00
C3—C4—C5	122.68 (14)	C3—C4—H4	119.00
O3—C5—C4	122.79 (13)	C5—C4—H4	119.00
N1—C5—C4	113.42 (13)	C6—C7—H7	120.00
O3—C5—N1	123.79 (13)	C8—C7—H7	120.00
C7—C6—C11	119.37 (13)	C7—C8—H8	120.00
N1—C6—C7	122.98 (14)	C9—C8—H8	120.00
N1—C6—C11	117.58 (13)	C9—C10—H11	120.00
C6—C7—C8	119.98 (14)	C11—C10—H11	120.00
C7—C8—C9	119.67 (14)	C6—C11—H12	120.00
N2—C9—C10	119.33 (14)	C10—C11—H12	120.00
N2—C9—C8	119.49 (13)		
			
C1—O1—C2—O2	4.0 (2)	C3—C4—C5—O3	4.5 (2)
C1—O1—C2—C3	178.82 (13)	C3—C4—C5—N1	−176.21 (14)
C5—N1—C6—C7	17.9 (2)	N1—C6—C7—C8	175.40 (14)
C5—N1—C6—C11	−165.10 (14)	C11—C6—C7—C8	−1.6 (2)
C6—N1—C5—O3	4.5 (2)	N1—C6—C11—C10	−175.18 (13)
C6—N1—C5—C4	−174.85 (13)	C7—C6—C11—C10	2.0 (2)
O5—N2—C9—C10	−12.0 (2)	C6—C7—C8—C9	−0.2 (2)
O5—N2—C9—C8	165.51 (14)	C7—C8—C9—N2	−175.78 (13)
O4—N2—C9—C10	169.43 (14)	C7—C8—C9—C10	1.7 (2)
O4—N2—C9—C8	−13.0 (2)	N2—C9—C10—C11	176.17 (13)
O1—C2—C3—C4	90.27 (17)	C8—C9—C10—C11	−1.3 (2)
O2—C2—C3—C4	−94.83 (19)	C9—C10—C11—C6	−0.5 (2)
C2—C3—C4—C5	1.5 (2)		

Symmetry codes: (i) −*x*+1, −*y*+1, −*z*; (ii) −*x*+1, −*y*+2, −*z*+1; (iii) −*x*+1, −*y*+2, −*z*; (iv) *x*−1, *y*, *z*; (v) −*x*, −*y*+1, −*z*; (vi) *x*, *y*−1, *z*−1; (vii) −*x*+2, −*y*+2, −*z*; (viii) *x*+1, *y*, *z*; (ix) −*x*, −*y*+2, −*z*+1; (x) *x*, *y*+1, *z*+1.

### Hydrogen-bond geometry (Å, °)

**Table:**

*D*—H···*A*	*D*—H	H···*A*	*D*···*A*	*D*—H···*A*
N1—H1···O2^viii^	0.86	2.11	2.9467 (17)	164
C7—H7···O3	0.93	2.33	2.8983 (17)	119
C11—H12···O3^viii^	0.93	2.45	3.3020 (19)	152

Symmetry codes: (viii) *x*+1, *y*, *z*.
